# Effect of Cell Concentration on the Persistence in the Human Intestine of Four Probiotic Strains Administered through a Multispecies Formulation

**DOI:** 10.3390/nu11020285

**Published:** 2019-01-29

**Authors:** Valentina Taverniti, Ranjan Koirala, Alessandro Dalla Via, Giorgio Gargari, Elena Leonardis, Stefania Arioli, Simone Guglielmetti

**Affiliations:** Division of Food Microbiology and Bioprocesses, Department of Food, Environmental and Nutritional Sciences (DeFENS), University of Milan, 20133 Milan, Italy; ranjan.koirala@unimi.it (R.K.); alessandro.dallavia@unimi.it (A.D.V.); giorgio.gargari@unimi.it (G.G.); elena.leonardis@studenti.unimi.it (E.L.); stefania.arioli@unimi.it (S.A.)

**Keywords:** *Bifidobacterium*, *Lactobacillus*, qPCR, viable recovery, dosage, strain-specific primers, healthy adults, intervention study

## Abstract

Studies devoted to evaluating the outcome of different doses of probiotics are very limited, especially for multistrain formulations. In this context, we performed an intervention study that aimed to compare the effect of the administration of two doses (7 billion and 70 billion bacterial cells) of a multistrain probiotic formulation on the persistence of the four probiotic strains that were present in the product in the fecal samples collected from healthy subjects. The overall persistence of the probiotic strains was significantly higher for the 70 billion formulation than for the 7 billion formulation. Furthermore, probiotic strains were detected earlier and for longer for the 70 billion formulation compared to those for the 7 billion formulation. All probiotic strains were recovered alive from the 70 billion preparation, whereas recovery was not possible in a few fecal samples upon administration of the 7 billion preparation. In addition, the overall number of viable probiotic cells recovered on day 14 (i.e., the last day of consumption) was significantly higher for the 70 billion formulation than that for the 7 billion formulation. Finally, we found that the viability of the probiotic cells was stable over the course of the trial independent of volunteers’ handling, demonstrating good manufacturing of the product. In conclusion, this study demonstrated that strains belonging to different taxa may coexist in the human gastrointestinal tract upon ingestion of a multispecies probiotic formulation. Moreover, this study suggests that higher doses of bacterial cells in probiotic formulations may permit a higher, earlier, and longer recovery of the probiotics in the feces of healthy adults.

## 1. Introduction

The ability to survive in the gastrointestinal environment is recognized as a fundamental requisite for probiotics [1], i.e., biologically active components of foods and food supplements defined as “live microorganisms that, when administered in adequate amount, confer a health benefit to the host” [2]. An appropriate survival rate in the gastrointestinal system is, in fact, considered mandatory for the performance of the metabolic activities that support probiotics’ health-promoting effects, such as the production of bacteriocins, organic acids, vitamins or, more generally, the exertion of competitive exclusion toward potential pathogens [3].

In 2001, an Expert Consultation of international scientists working on behalf of the Food and Agriculture Organization of the United Nations (FAO) and the World Health Organization (WHO) stated that “the ability to remain viable at the target site should be verified for each potentially probiotic strain” [1]. However, the possibility of withstanding gastrointestinal transit (GITr) depends not only on the intrinsic properties of the microbial strain but also on the number of viable cells administered and, therefore, on the manufacturing procedures adopted, which must guarantee the maintenance of a proper viable count of probiotic cells until the end of the shelf life of the product. Reportedly, probiotic viability throughout storage period of commercial products can be poor (for a review see: [4]). In fact, several studies have reported a reduction in viable cells per dose below the limit stated on the label and below the recommended minimum dose for a potential effect on the host, which was established to be one billion colony-forming units (CFUs) [5]. The decline in microbial viability over the shelf life of the product highlights the importance of selecting shelf-stable probiotic strains, excipients with a low water activity and appropriate packaging. Consequently, proof of survival in the gut should not only be assessed for a particular probiotic strain but also for each specific commercial product that contains such probiotic microorganism.

The assessment of survival during GITr and the persistence of a probiotic microorganism in the gut can be carried out through fecal recovery studies, which are human intervention trials consisting of the quantification and/or reisolation of a specific probiotic strain in feces after oral administration. To date, very few studies have documented the survival of probiotics during GITr [6,7,8,9], and such investigation has not been carried out for most products available on the market. Particularly, recovery studies are missing for multistrain probiotic formulations, for which possible inter-microbe interference can also be postulated.

Based on these observations, our study aimed to compare the impact of two doses of a four-strain probiotic product on the ability of each strain to withstand GITr and persist in the gut of healthy adults. To this aim, cultural methods have been combined with molecular protocols based on strain-specific primers to quantify bacterial cells and assess their viability in fecal samples collected from healthy adult volunteers during the two-week probiotic administration period and during two weeks of follow-up. Finally, the stability of bacterial cultures in the product was assessed by means of flow cytometry analyses of capsules returned by the volunteers at the end of the study.

## 2. Materials and Methods

### 2.1. Human Intervention Study

#### 2.1.1. Study Population

The study “Effect of cell concentration on permanence of four probiotic strains in the human intestine (ECoCePPI study)” was designed as a single-blind, two-arm parallel microbiological pilot study. Regarding participants, 20 volunteers per arm (40 volunteers in total Supplementary Table S1) were enrolled by selecting healthy (nondiseased) adult volunteers recruited according to the criteria described below.

Inclusion criteria. Volunteers of both sexes aged between 18 and 60 who signed informed consent documents.

Exclusion criteria. (i) Antibiotic consumption in the month prior to the beginning of the project; (ii) consumption of antacids or prokinetic drugs; (iii) chronic intestinal inflammatory diseases; (iv) infectious intestinal diseases; (v) viral or bacterial enteritis episodes within the 2 months preceding the study; (vi) episodes of gastric or duodenal ulcers in the previous 5 years; (vii) pregnant or breastfeeding; (viii) recent history or suspected abuse of alcohol or drugs; (ix) any severe disease that may interfere with the treatment; (x) insufficient reliability or presence of conditions that may cause a volunteer’s nonconformity/adherence to the protocol; and (xi) previous participation in this study. 

#### 2.1.2. Formulations under Study

The probiotic formulations (containing shelf-stable industrially prepared lyophilized cells) were provided by GIELLEPI S.p.A. (Lissone, Italy), which produced them following internal industrial procedures. The 70 billion preparation is available on market in Italy with the commercial name “FlorMidabìl™” The formulations consisted of methylcellulose capsules containing a blend of four probiotic strains: *Bifidobacterium animalis* subsp. *lactis* Bl-04 (human fecal origin), *Lactobacillus acidophilus* La-14 (human fecal origin), *Lactobacillus plantarum* SDZ-11 (from fermented vegetables) and *Lactobacillus paracasei* SDZ-22 (dairy origin) at two doses: 70 billion (Formulation 1) and 7 billion (Formulation 2) CFUs/capsule. Bacterial strain concentrations and excipients are indicated in Table 1.

#### 2.1.3. Intervention and Study Scheme

**Figure 1:**
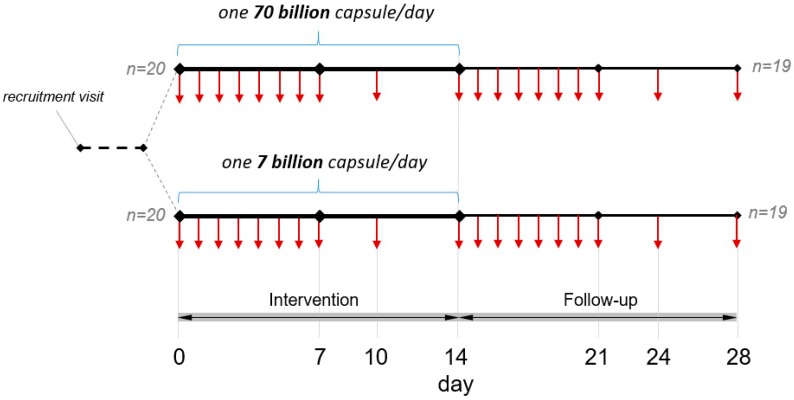
Schematic of the study design. Vertical arrows indicate the days of fecal sample collection (when defecation occurred) from volunteers.

Eligible participants were invited to a detailed explanation of the study. Candidates willing to participate signed an informed consent document, were instructed on the procedures for the storage and transportation of the fecal samples and were provided with all material necessary for the study, including 14 + 2 extra capsules of the probiotic product; 19 plastic jars for the collection of all fecal samples (each one marked with the number of the respective collection day); disposable plastic bags for the transportation of the jars; a calendar with each day of fecal sample collection highlighted; a detailed protocol with all the information provided to properly follow the study time-line; and daily questionnaires to be filled out for all 28 days of the study. Volunteers had to report daily bowel habits according to the Bristol Stool Chart. In addition, volunteers were asked to report symptoms related to meteorism, flatulence, and nausea and any other anomalous adverse or positive symptoms weekly. Forty subjects were recruited (27 females and 13 males) and randomized into two arms with 20 subjects each: arm A (from Subject S01 to S20, mean age 29.2 ± 10.5; 15 females and 5 males) received the 7 billion dose, whereas arm B (from S21 to S40, mean age 29.1 ± 8.6; 12 females and 8 males) received the 70 billion dose. The study entailed 2 weeks of probiotic consumption and 2 weeks of follow-up (Figure 1). For the entire duration of the trial, volunteers were asked to follow their usual diet, with the only recommendation to avoid intake of other probiotic products, either as fermented dairy products or as food supplements. Consumption of traditional yogurt was allowed. Volunteers were instructed to consume the probiotic product in the morning, at least half an hour before breakfast, or in the evening, at least 2 h after the meal and to keep the probiotic capsules at room temperature. At the end of the study, volunteers returned the filled-in questionnaires and the two extra probiotic capsules to the laboratory.

#### 2.1.4. Sample Collection

Volunteers were asked to collect, assuming compatibly with bowel habits, 19 fecal samples in total, in accordance with the scheme reported in Figure 1: the first fecal sample on day 0 corresponds to the day before starting supplementation with the probiotic formulation; samples from day 1 to 7 refer to a daily fecal collection to be done during the first week of probiotic product consumption; collections on day 10 and 14 were performed to obtain samples from an intermediate point and at the end of the probiotic supplementation period, respectively. Samples from days 15 to 21 corresponded to a daily fecal collection during the first week of the follow-up, whereas samples from days 24 and 28 refer to the middle and the end of the follow-up phase, respectively. According to the instructions provided to volunteers, all the fecal samples were maintained at 4 °C for a maximum of 72 h until delivery to the laboratory, where the samples were stored at −80 °C until DNA extraction. Samples from day 14 were delivered to the laboratory within 24 h and were immediately processed for viable recovery.

#### 2.1.5. Ethical Statement

The study protocol was approved by the Research Ethics Committee of the University of Milan (opinion no. 50/17, 18 December 2017). Written informed consent was obtained from all subjects before recruitment. 

#### 2.1.6. Volunteer Compliance

Volunteer compliance was determined by verbal assessment and count of the capsules returned by each volunteer at the end of the study.

### 2.2. DNA Extraction from Fecal Samples

All fecal samples obtained from a single subject were thawed and processed at the same time. After homogenization, 250 mg of feces were weighed and processed using a DNeasy PowerLyzer PowerSoil kit (Qiagen, Hilden, Germany) for the isolation of total DNA with a minor modification that included incubating samples at 65 °C for 10 min after the addition of the power bead and C1 solutions provided with the kit. Steps of mechanical bacterial cell disruption were performed using a Precellys bead beater (Advanced Biotech Italia s.r.l., Seveso, Italy) that was kept in a cold room (3 cycles of 6800 rpm × 30 s). Afterwards, extractions were performed according to the manufacturer’s instructions. After isolation, DNA was quantified, and the 260/280 ratio was measured with a Take3 Micro-Volume analyzed in a microplate reader using Gen5 software (BioTek Instruments, Inc., Winooski, VT, USA). After dilution, DNA samples were stored at −80 °C until the analyses.

### 2.3. Quantification of Bacterial Strains through Quantitative Polymerase Chain Reaction (qPCR)

The four bacterial strains under investigation were quantified by means of the species/strain-specific primers reported in Table 2. Primer sets for the quantification of *B. animalis* subsp. *lactis* Bl-04, *L. plantarum* SDZ-11 and *L. paracasei* SDZ-22 were developed and provided by Danisco S/A (DuPont). Strain-specific primers for the detection of *L. acidophilus* La-14, which were developed in this study, were designed in a region of 416 bp known to be deleted in *L. acidophilus* strains that share high genome similarity with La-14 [10]. The validation of primers, preparation of calibration curves, and qPCR conditions are described in Appendix A. Quantitative PCR (qPCR) with strain-specific primers was used to enumerate the four bacterial strains under study in the feces that was delivered by the volunteers over the two weeks of probiotic intake and during the two-week follow-up according to the scheme reported in Figure 1.

### 2.4. Viable Recovery of Probiotic Strains

Fecal samples from day 14, corresponding to the last day of the probiotic consumption (the first subsequent evacuation available was considered for a few samples when no evacuation occurred on the 14th day), were processed to verify the ability of the four probiotic strains to survive gastrointestinal transit. For this purpose, one gram of fecal sample was diluted in Maximum Recovery Diluent (Oxoid, Basingstoke, UK), homogenized in a sterile stomacher bag by using a Colworth Stomacher 400 instrument (Seward, West Sussex, UK) and plated on MRS supplemented with 0.05% cysteine and 5 µg mL^−1^ streptomycin (scMRS). At least five dilutions per fecal sample were plated. After 48 h of incubation at 37 °C in anaerobic conditions with the use of Anaerocult A (Merck, Kenilworth, NJ, USA), all the colonies from each dilution plate were collected separately, and the biomass from the respective dilution plates was used for total DNA isolation as described above. Afterwards, qPCR with probiotic-specific primers was performed on the DNA isolated from the colony biomasses. The highest dilution giving a positive signal in qPCR and the obtained Cq value were used to calculate the “estimated minimum CFU number” (eCFU) for each investigated strain.

### 2.5. Assessment of Bacterial Cell Stability in Probiotic Capsules throughout the Study

Product stability was analyzed in terms of the total number of viable probiotic cells in the capsules returned by the volunteers at the end of the trial. Specifically, total viability was evaluated by flow cytometry according to a modified ISO 19344 IDF 232 procedure (protocol B), using SYTO 24 (Thermo Fisher Scientific, Rodano, Italy) and propidium iodide (PI) (Sigma-Aldrich, Milan, Italy) as staining solutions. SYTO 24 permeates the membrane of all cells and binds the nucleic acids, making cells fluorescent green. PI penetrates only bacteria with damaged membranes, causing a reduction in SYTO 24 fluorescence when both dyes are present. Thus, live bacteria with intact cell membranes fluoresce bright green (defined as Active Fluorescent Cells), bacteria with slightly damaged membranes exhibit both green and red fluorescence (defined as damaged cells), whereas bacteria with broken membranes fluoresce red (defined as non-Active Fluorescent Cells).

The viability was assessed by dissolving powder from the two left over capsules in Mitsuoka buffer (pH 6.5), homogenizing the solution in a sterile stomacher bag by using a Colworth Stomacher 400 instrument (Seward, West Sussex, UK), serially diluting the solution in the same buffer, and staining with SYTO 24 and PI in the dark at 37 °C for 15 min. After the dual staining procedure, samples were immediately analyzed by a C6 BD Accuri™ flow cytometer (BD Biosciences, San Jose, CA, USA) using the following threshold settings: FSC-H: 5000 and SSC-H 2000; total volume collected, 50 µL. All the parameters were collected as logarithmic signals. A 488 nm laser was used to measure the FSC values. The data were analyzed using BD Accuri™ C6 software version 1.0 (BD Biosciences, Milan, Italy). The SYTO 24 fluorescence intensities of stained cells were recovered in the FL1 channel (excitation, 488 nm; emission filter, 530/30; BD Biosciences). The PI fluorescence was recovered in the FL3 channel (excitation, 488 nm; emission filter, 610/20; BD Biosciences, Milan, Italy). The absolute flow cytometry count was performed using Fluoresbrite polychromatic red 2.0 mm microspheres as a reference. An example of a flow cytometry measurement dot plot of the stained product is shown in Supplementary Figure S1.

### 2.6. Statistical Analysis

Statistical calculations were performed using the software program GraphPad Prism 5. The significance of differences was assessed by unpaired Student’s *t*-test with two-tailed distribution. Differences of *p* < 0.05 were considered significant. 

## 3. Results

### 3.1. Adherence to Study Protocol and Case Reports

The compliance of the volunteers was excellent since, out of the 40 subjects recruited, 38 completed the study as per the protocol in terms of probiotic consumption and fecal sample collections; specifically, one subject from arm A (7 billion dose) dropped out on day 14 (the last day of the probiotic consumption), and one participant from arm B (70 billion dose) dropped out of the study on day 21, both due to antibiotic consumption. One subject from arm A did not return the questionnaire, whereas 4 subjects (3 from arm A and 1 from arm B) did not return the extra capsules. Two subjects out of the 19 subjects in arm A and 1 out of the 20 subjects in arm B reported improved regularity regarding bowel habits. One subject from arm A and 7 subjects from arm B reported episodes of meteorism or flatulence. One subject from arm B reported gastro-esophageal reflux, and another subject from arm B reported abdominal discomfort. One volunteer from arm B noted an amelioration of dermatitis. Throughout the study period, evacuation frequency or consistency (according to the Bristol Stool Chart) did not change significantly (Supplementary Figure S2).

### 3.2. Enumeration of Probiotic Bacteria in Fecal Samples

qPCR analyses were performed on the four strains for each subject (Supplementary Figure S2). *B. animalis* subsp. *lactis* Bl-04 was detected in at least one fecal sample from all subjects in both randomized arms (lower and higher doses). In contrast, *L. acidophilus* La-14 was detected in all 20 subjects who received the 70 billion dose and in 18 out of the 20 subjects who received the 7 billion dose. Additionally, *L. plantarum* SDZ-11 was found in the feces of all volunteers in the 70-billion arm, whereas it was detected in only 15 of the 20 subjects in the 7-billion arm. Finally, *L. paracasei* SDZ-22, which was the strain with the lowest concentration in the product, was detected in 18 out of the 20 subjects who received the 70 billion dose, whereas it was under the detection limit (4.5 log10 cells per g of feces) in most subjects in the 7-billion arm (SDZ-22 was detected in only 4 subjects) (Supplementary Figure S2).

**Figure 2:**
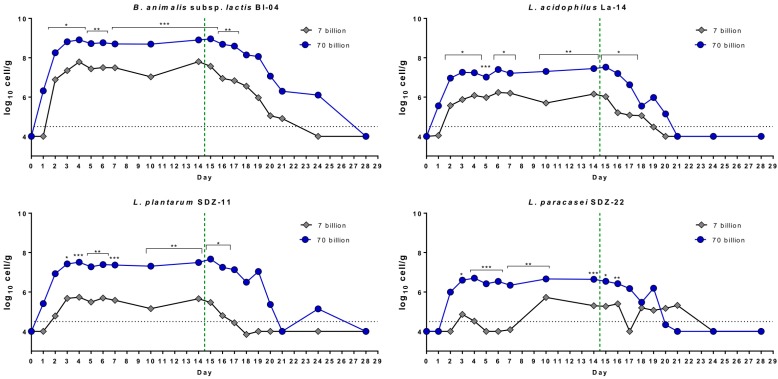
Mean recovery per day of the four probiotic strains simultaneously administered to volunteers during the ECoCePPI study with a multistrain formulation at two doses. Data come from qPCR experiments with strain-specific primers. Statistics according to two-tailed unpaired *t*-test; *, *p* < 0.05; **, *p* < 0.01; ***, *p* < 0.001.

**Figure 3:**
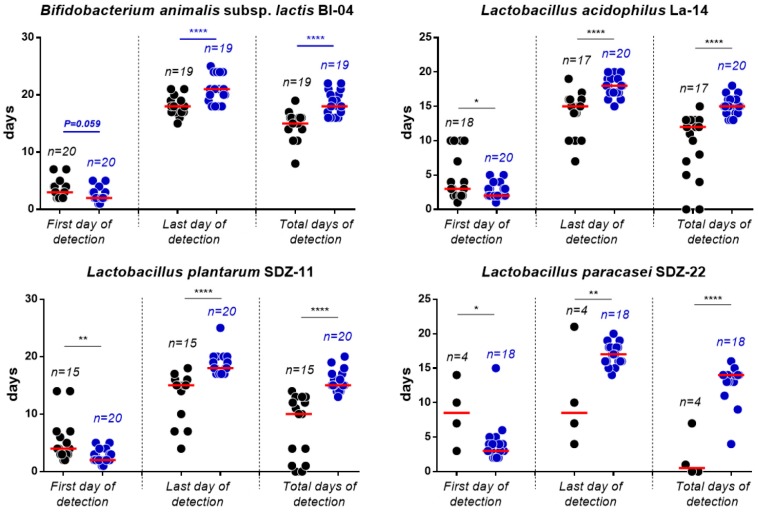
First, last, and total days of detection of probiotic strains in the fecal samples of volunteers receiving the 7 billion (black circles) and 70 billion (blue circles) formulations as demonstrated by qPCR analyses. Statistical analysis was performed according to a two-tailed unpaired *t*-test; *, *p* < 0.05; **, *p* < 0.01; ****, *p* < 0.0001.

Overall, we quantified a higher number of bacterial cells for all strains, both during the treatment phase and the follow-up in fecal samples collected from the group receiving the 70 billion dose, according to two-tailed unpaired *t*-test (Figure 2). Moreover, all strains were detected earlier and for longer in the feces of the participants who received the 70 billion formulation. Specifically, in the fecal samples of the volunteers who received the 70 billion formulation, the detection of probiotic strains occurred approximately 1 day earlier, ended 3 days later, and resulted in a 3-day longer detection period (statistics based on unpaired *t*-test with two-tailed distribution; Figure 3). This difference was particularly evident in the case of *L. paracasei* SDZ-22, which, as reported above, is the strain present in the lowest concentration in the product (Figure 3).

### 3.3. Viable Recovery of Probiotic Strains from Fecal Samples

**Figure 4:**
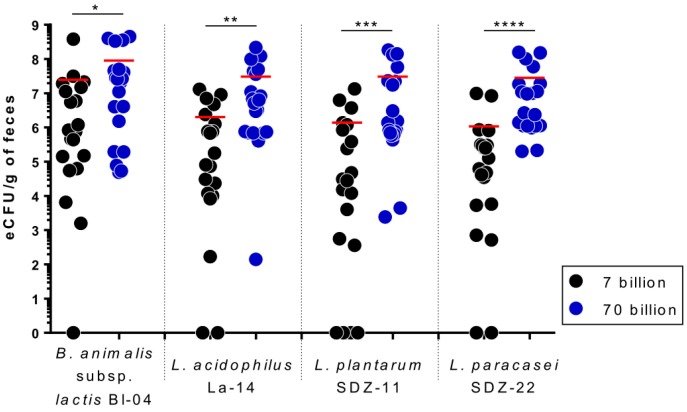
Recovery of viable cells of the probiotic bacterial strains from the feces collected at the end of the two-week intervention (day 14). Data are reported as the estimated minimum CFU number (eCFU; see methods section in the text for details). Statistical analysis was performed according to a two-tailed unpaired *t*-test; *, *p* < 0.05; **, *p* < 0.01; ***, *p* < 0.001; ****, *p* < 0.0001.

The fecal samples collected on the last day of probiotic consumption (or the first evacuation on a subsequent day, if evacuation did not occur on day 14) were analyzed to assess the GITr transit survival of the four probiotic strains under study. We recovered viable cells of Bl-04, La-14, SDZ-11, and SDZ-22 from all the volunteers consuming the 70 billion dose, whereas in the 7-billion arm, the recovery was not successful for 5 subjects: S06 and S11 were negative for SDZ-11; S12 was negative for SDZ-11, La-14, and Bl-04; S14 was negative for La-14 and SDZ-22; and S15 was negative for La-14 and SDZ-22 (Supplementary Figure S3). Moreover, the viable recovery was significantly higher after 2 weeks of consumption of the 70 billion probiotic preparation in comparison with that of the 7-billion formulation (Figure 4).

### 3.4. Stability of the Probiotic Bacteria in Capsules during the Trial

Capsules of both probiotic formulations returned from the volunteers at the end of the study were used to quantify the total viable bacterial cells. Data were compared with the viable counts of capsules stored under proper conditions in the laboratory. We observed that the different storage conditions associated with the volunteers’ diverse handling of the probiotic product did not have any significant impact on stability in terms of viability of the probiotic cells. Specifically, flow cytometry analyses showed that there were no significant differences between capsules handled by volunteers and those properly stored in the laboratory, both in the case of the 7 and 70 billion formulations. We only found reduced viability in the capsules returned from subject S36 (arm B, 70-billion dosage), where the viable count was approximately 1 log lower than the average of the other capsules (Supplementary Figure S4). In this capsule, we found signs of powder clogging.

## 4. Discussion

The markers useful in determining the potential effects of functional foods on the health of nondiseased people have been classified into three categories: (a) markers related to the exposure of the host to the food component under study; (b) markers associated with a specific physiological function or biological response; and (c) markers associated with a general health condition (e.g., reduced risk of disease) [11]. The demonstration of the survival of a probiotic strain in the gastrointestinal tract and its detection in feces after a specific time (i.e., recovery and persistence studies) can be classified as type (a) markers [12], whose assessment has been explicitly suggested to be a requisite for each probiotic strain [2,13]. However, the ability to survive and persist in the gastrointestinal tract has been shown only for a limited number of probiotics available on market and is mostly limited to the well-known probiotic strains *Lactobacillus rhamnosus* GG [9,14], *Lactobacillus paracasei* Shirota [7,15], *Bifidobacterium animalis* subsp. *lactis* Bb12 [16,17] and *Lactobacillus paracasei* DG [6]. Furthermore, recovery studies reported in the literature mostly relate to single-strain probiotic products. However, although it has not been scientifically demonstrated whether a combination of strains is more efficacious than a single strain, the number of commercially available multistrain probiotic formulations is increasing, supported by studies suggesting the existence of synergistic effects between different probiotic strains that improve effectiveness compared to the strains used singularly [18,19,20].

Contextually, in the study presented here, we aimed to evaluate the influence of dose (i.e., cellular concentration) on the ability of four probiotic strains administered once daily through a multistrain product in capsule to persist in healthy adult intestines. The commercial bacterial blend used in this study has been selected because it includes bacterial strains belong to the classes of microorganism most commonly used as probiotics by industry, i.e., bifidobacteria (strain Bl-04), homofermentative lactobacilli (La-14), and hetero-fermentative lactobacilli (SDZ-22 and SDZ-11).

The results obtained demonstrated that all strains investigated can be recovered from feces, maintaining approximately the relative proportion they had in the product, suggesting that in the gastrointestinal tract there is no prevalent growth of a strain compared to the others, even though it could be expected that gut environmental conditions might affect differently bacteria depending on their physiological characteristics. In accordance with our results, a recent study carried out in an in vitro colon simulator showed that the detection level of four probiotic strains was not affected when the strains were administered alone or simultaneously through a multistrain product [21]. Therefore, the postulated hypothesis that probiotics may be antagonistic toward each other when in the same formulation and/or in the gastrointestinal tract, although not thoroughly investigated, seems to be questionable, or at least needs to be verified on a case-by-case basis.

Although a definitive demonstration of an adequate (optimal) number of probiotic cells to be consumed to confer an effect on the host has not been performed, it is considered plausible that the efficacy of a probiotic microorganism can be enhanced as it remains alive in the host’s intestine longer and in higher concentrations. Accordingly, supplementation with a multispecies probiotic showed improved beneficial effects on the cardiometabolic parameters and gut permeability of obese postmenopausal women when administered as a high dose (1 × 10^10^ CFU/day) compared to a low dose (2.5 × 10^9^ CFU/day) formulation [22]. In another study, a probiotic product containing a single *Lactobacillus gasseri* strain significantly reduced the abdominal pain score in irritable bowel syndrome (IBS) patients when administered at a high dose (10^10^ CFU/day) but not at a low (2 × 10^8^ CFU/day) or intermediate (2 × 10^9^ CFU/day) dose [23]. Again, abdominal pain and bloating associated with antibiotic use in a hospital setting were found to be reduced upon administration of a four-strain probiotic product at a high dose (1.70 × 10^10^ CFU/day) compared to the same blend at a lower dose (4.17 × 10^9^ CFU/day) [24]. Notably, the bifidobacterial strain used in this trial (*B. animalis* subsp. *lactis* Bl-04) is the same as the probiotic formulations used in our study. In this context, our study showed that a higher dosage of probiotics allows not only a greater recovery of live cells in the intestine but also an earlier detection and longer permanence, plausibly promoting a more rapid and longer-lasting efficacy.

The delivery of an adequate number of live cells into the host’s intestine is unavoidably related to the conservation of viability of probiotic cells in the product. Although available industrial technologies may guarantee an excellent survival of probiotic microorganisms in the appropriately stored product, little is known about the fate of microbial cells once the probiotic product is opened and handled by the consumer. For this reason, in this study, we evaluated the viability of the probiotic bacteria within the product at the end of the study, i.e., following the usage and conservation of the capsules by each volunteer. Interestingly, the collected data showed excellent microbiological stability of the product, with the maintenance of bacterial viability at the levels of capsules properly stored in the laboratory, with the only exception of one sample that showed biomass clogging as a possible result of inadequate product handling. Therefore, these data demonstrate the importance of good probiotic product manufacturing (including the selection of proper packaging) to overcome the impact that consumers’ handling may have on microbial cell viability inside the product.

## 5. Conclusions

In conclusion, this study demonstrated that different strains belonging to diverse taxa may effectively be employed together and be selectively quantified upon ingestion through a multispecies probiotic formulation. Moreover, we highlight that higher doses of bacterial cells in probiotic formulations may allow a higher, earlier, and longer recovery of the probiotic strains in the feces of healthy adults.

## Figures and Tables

**Table 1:** Composition of probiotic capsules used in the ECoCePPI study.

Ingredient	Formulation 1	Formulation 2
Coating Agent	Hydroxypropyl methylcellulose	Hydroxypropyl methylcellulose
Bulking agent	Microcrystalline cellulose	Microcrystalline cellulose
Anti-caking agent	Magnesium salts of fatty acids and silicon dioxide	Magnesium salts of fatty acids and silicon dioxide
Coloring agent	Titanium dioxide	Titanium dioxide
**Probiotic strains** (lyophilized biomass; 10^9^ CFUs/capsule)		
*Bifidobacterium animalis* subsp. *lactis* BL-04	5.25	52.5
*Lactobacillus acidophilus* LA-14	1.4	14
*Lactobacillus plantarum* SDZ-11	0.28	2.8
*Lactobacillus paracasei* SDZ-22	0.07	0.7
**Total probiotic cells**	7	70

**Table 2:** Primer sets used for the qPCR experiments in the ECoCePPI study.

Primer Name	Primer Sequences	Target Strain	Source
Bl-04-F Bl-04-R	5′-CTTCCCAGAAGGCCGGGT-3′ 5′-CGAGGCCACGGTGCTCATATAGA-3′	*B. animalis* subsp. *lactis* BL-04	[25]
La-14-F La-14-R	5′-ATCGTGATTTGCATATAAATTGA-3′ 5′-ACCTTGCTTAATTTGCAAGTCT-3′	*L. acidophilus* LA-14	This study
SDZ-11-F SDZ-11-R	5′-CTTGATGACTCTTCTGGGGC-3′ 5′-ACGGGAGTGATAGACGTTGAG-3′	*L. plantarum* SDZ-11	This study
SDZ-22-F SDZ-22-R	5′-TTGGGTGCTATGGGAAACACA-3′ 5′-AAGTTACGCCGCCACAAAC-3′	*L. paracasei* SDZ-22	This study
357F 907R	5′-CCTACGGGAGGCAGCAG-3′ 5′-CCGTCAATTCMTTTRAGTTT-3′	Panbacteria	[26]

## Supplementary Materials

The following are available online at http://www.mdpi.com/2072-6643/11/2/285/s1, Figure S1: Representative biplot from flow cytometry analysis of the bacterial cells stained with SYTO 24 and propidium iodide in the probiotic capsules at 7 (panel A) and 70 (panel B) billion dosages. Active Fluorescent Units (AFU) were identified in the green gate (presumed live cells), damaged cells were identified in orange gate, non-Active Fluorescent Units (nonAFU) were identified in the red gate (presumed dead cells). Channels FL1 and FL3 used the measurement of fluorescence are described in the text, Figure S2: Detection of the probiotic strains along the ECoCePPI study reported for each subject from the 7-billion (subjects S01 to S20) and the 70-billion (S21 to S40) arm. Quantification of probiotic strains in fecal samples through qPCR is reported as number of cells expressed in log10 cells/g of feces (left Y axis). Gray histograms refer to the number of evacuations, whereas blue histograms refer to the Bristol stool scale (both on the right Y axis) as reported daily by volunteers in the questionnaire. Bl-04: *B. animalis* subsp. *lactis* Bl-04; La-14: *L. acidophilus* La-14; SDZ-11: *L. plantarum* SDZ-11; SDZ-22: *L. paracasei* SDZ-22. The horizontal dotted gray lines indicate the detection limit of bacterial cell quantification in qPCR; Figure S3: Isolation of viable cells of the probiotic strains from the feces collected at the end of the intervention period (day 14). Positive recovery is indicated in green. Red boxes indicate that viable cells of a specific probiotic strain have not been isolated from the feces of a specific subject. Figure S4: Stability of probiotic cell viability in capsules during the ECoCePPI study as determined through flow cytometer experiments. More details on the flow cytometer protocol adopted are available in the text (material and methods section) and in Figure S1. Table S1: Basic characteristics of study participants.

## Appendix A. Validation of Strain- and Species-Specific Primers and Preparation of Calibration Curves for Bacterial Quantification through qPCR

All primer sets were tested in gradient PCR to find the most suitable annealing temperature. Reactions were performed on a Bio-Rad CFX96 system with the use of SYBR-Green technology. The reaction mix was prepared in a final volume of 15 µL containing 7.5 µL of EvaGreen Supermix (Bio-Rad Laboratories, Segrate, Italy), 0.5 μM of each primer pair, and 10 ng of the target bacterial DNA. The thermal cycle was as follows: 3 min at 95 °C, followed by 39 cycles of 10 s at 95 °C, a temperature ramp from 54 °C to 62 °C for 0.05 s, followed by an extension at 72 °C for 10 s. Then, primer pairs Bl-04 F-R, La-14 F-R, SDZ-11-F/R and SDZ22-F/R were checked for strain specificity by testing the potential amplification of the DNA isolated from at least 3 other strains belonging to the species *B. animalis*, *L. acidophilus*, *L. plantarum* and *L. paracasei*, respectively. Primer efficiency was calculated by testing all the primer pairs at concentrations of 0.3, 0.4 and 0.5 μM in serial 1:3 dilutions of the corresponding microbial target DNA extracted from pure bacterial cultures of Bl-04, La-14, SDZ-11, and SDZ-22. All primer sets gave no amplification on other strains belonging to the same species of target strains, with the only exception of primers for *L. plantarum* SDZ-11 that gave a signal for a noncommercial *L. plantarum* strain. The four primer pairs were also tested on the fecal samples of all 40 subjects collected on day 0 to rule out the presence of a possible amplification signal not ascribable to probiotic consumption. No specific amplification was found on day 0, with the only exception of 3 subjects who exhibited an amplification signal with the same melting curve of *L. paracasei* SDZ-22. The total number of bacteria in fecal samples was quantified by using panbacterial primers 357F-907R targeting the V3-V5 region of the 16S rRNA gene (Table 2).

Calibration curves for the quantification of probiotic strains were set up by extracting DNA from a fecal mixture prepared by mixing representative T0 feces, selected according to the Bristol chart; then, an equal number of the four bacterial strains under study was added to the fecal mixture. For this purpose, bacterial cells of strains Bl-04, La-14, SDZ-11, and SDZ-22 were mixed and serially diluted starting from 1 × 10^9^ to 1 cell; each of the 10 mixed bacterial suspensions was added to an aliquot of the fecal mixture to mimic the extraction conditions from the fecal matrix. Subsequently, DNA was isolated from all 10 concentration mixtures using the kit reported above. After quantification, stocks of 10 ng of DNA per μL of each dilution were prepared for use in qPCR with the probiotic-specific primer pairs. To prepare calibration curves, the quantitation cycle (Cq) obtained in qPCR corresponding with each dilution was correlated with the known number of bacterial cells added to stool samples for extraction. The equation obtained from the calibration curve was then employed to calculate the number of bacterial cells in the DNA isolated from the fecal samples of the volunteers based on the obtained Cq value. All fecal DNA samples were tested by qPCR at a final amount of 50 ng in a volume of 15 µL containing 7.5 µL of EvaGreen Supermix and 0.3 µM of each primer. The cycling parameters used for La-14 and SDZ-11 were initiated for 3 min at 95 °C, followed by 39 cycles of 10 s at 95 °C, 30 s at 56 °C and 0.05 s at 72 °C using the Bio-Rad CFX96 system. For SDZ-22, the annealing T was set at 56.6 and 60 °C without an elongation step for the amplification of Bl-04. In each run, samples for the calibration curve and a negative (no DNA) and positive control (target bacterial DNA) were included. All samples were run in duplicate in each plate. Melting curves were analyzed with Bio-Rad CFX Manager 3.1 software to confirm the specificity of the amplification products. The calibration curve for the quantification of the total bacteria present in fecal samples was prepared by using six bacterial strains belonging to the phyla Actinobacteria, Firmicutes, and Proteobacteria. Equal numbers of cells for each strain were mixed. DNA extraction was carried out from a total of 4 × 10^10^ cells using the previously mentioned kit. The mixed bacterial DNA obtained was quantified and diluted to various concentrations, taking into consideration the total number of cells used for DNA extraction and the amount of DNA yielded. qPCR amplification was carried out in a final volume of 15 µL containing 7.5 µL of EvaGreen Supermix and 0.5 µM of each primer (357F and 907R). Different amounts of DNA, i.e., ranging from 0.0013 to 13 ng (corresponding to 4 × 10^3^ to 4 × 10^7^ cells), were used in each reaction. The amplification was carried out using the following program: initial hold at 95 °C for 3 min and 39 cycles at 95 °C for 30 s, 58 °C for 30 s and 72 °C for 30 s. Melting curves were analyzed to confirm the specificity of the amplification products (Bio-Rad CFX Manager 3.1), and a standard curve was plotted with the Cq values and Log10 number of cells added with respect to the amount of DNA.
