# Supplementary material for: Effect of Cell Concentration on the Persistence in the Human Intestine of Four Probiotic Strains Administered through a Multispecies Formulation
Source: Nutrients. 2019 Jan 29;11(2):285. doi: 10.3390/nu11020285 (PMC6412360; doi:10.3390/nu11020285)

Table S1. Basic characteristics of study participants.

**Randomization group 1** (7 billion)			**Randomization group 2** (70 billion)		
**Subject** (n=20)	**Sex** (15F/5M)	**Age** (19-59 years)	**Subject** (n=20)	**Sex** (12F/8M)	**Age** (22-49 years)
S01	F	23	S21	M	23
S02	F	57	S22	F	32
S03	F	34	S23	F	24
S04	F	26	S24	F	22
S05	M	27	S25	M	25
S06	M	27	S26	M	24
S07	F	24	S27	F	35
S08	M	26	S28	F	24
S09	F	23	S29	M	29
S10	F	19	S30	F	24
S11	F	23	S31	F	43
S12	F	22	S32	M	28
S13	M	24	S33	F	23
S14	F	29	S34	F	23
S15	F	28	S35	M	23
S16	F	32	S36	F	35
S17	F	24	S37	M	48
S18	M	32	S38	F	24
S19	F	25	S39	F	23
S20	F	59	S40	M	49

Figure S1. Representative biplot from flow cytometry analysis of the bacterial cells stained with SYTO 24 and propidium iodide in the probiotic capsules at 7 (panel A) and 70 (panel B) billion dosages. Active Fluorescent Units (AFU) were identified in the green gate (presumed live cells), damaged cells were identified in orange gate, non-Active Fluorescent Units (nonAFU) were identified in the red gate (presumed dead cells). Channels FL1 and FL3 used the measurement of fluorescence are described in the text.

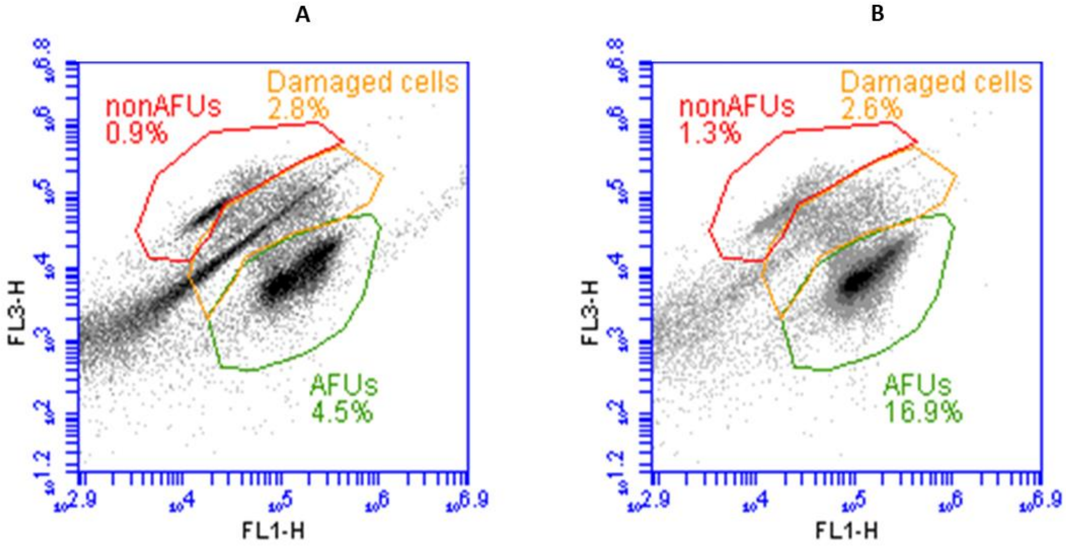

Figure S2. Detection of the probiotic strains along the ECoCePPI study reported for each subjects from the 7-billion (subjects S01 to S20) and the 70-billion (S21 to S40) arm. Quantification of probiotic strains in fecal samples through qPCR is reported as number of cells expressed in log10 cells/g of feces (left Y axis). Gray histograms refer to the number of evacuations, whereas blue histograms refer to the Bristol stool scale (both on the right Y axis) as reported daily by volunteers in the questionnaire. Bl-04: *B. animalis* subsp. *lactis* Bl-04; La-14: *L. acidophilus* La-14; SDZ-11: *L. plantarum* SDZ-11; SDZ-22: *L. paracasei* SDZ-22. The horizontal dotted gray lines indicate the detection limit of bacterial cell quantification in qPCR.

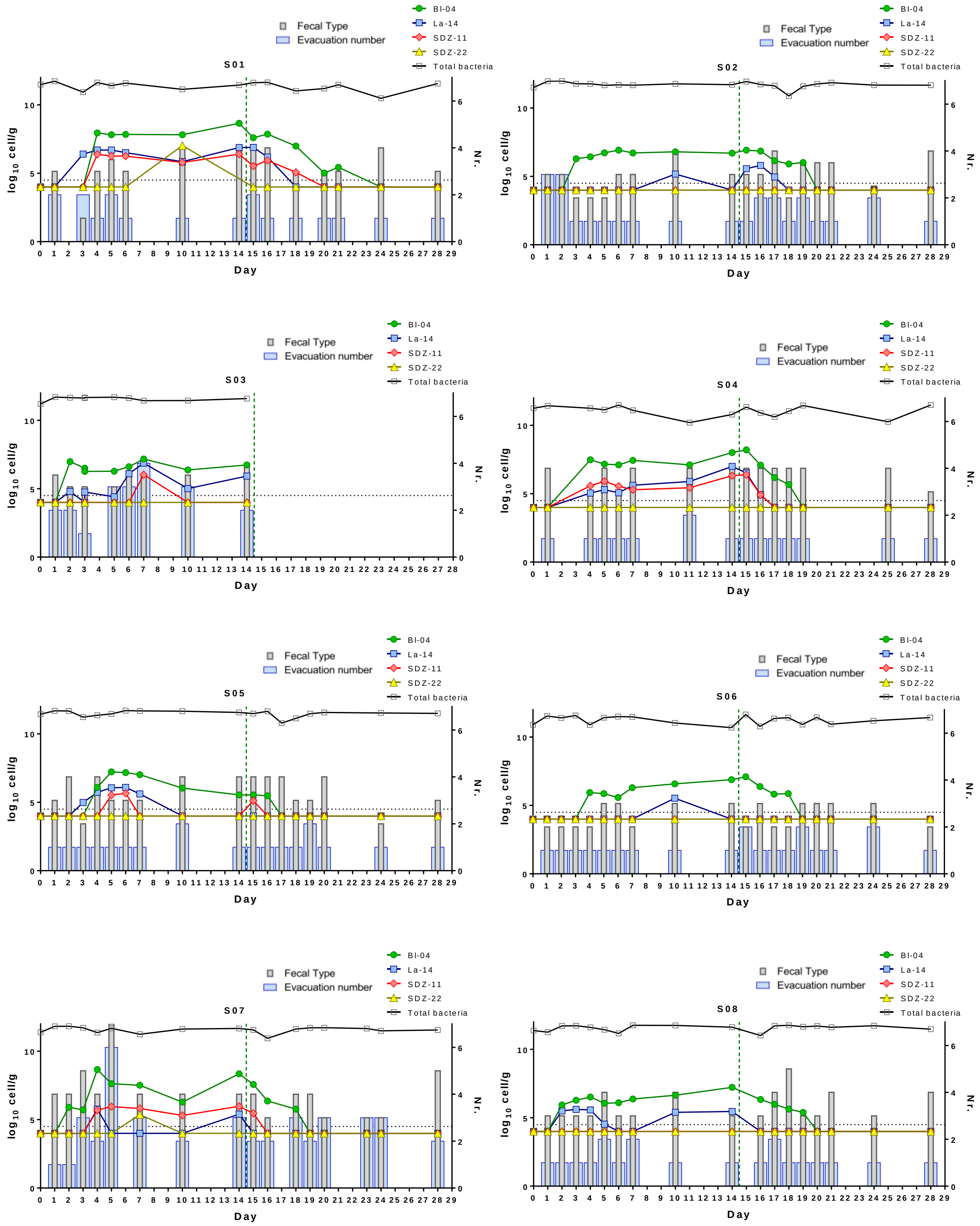

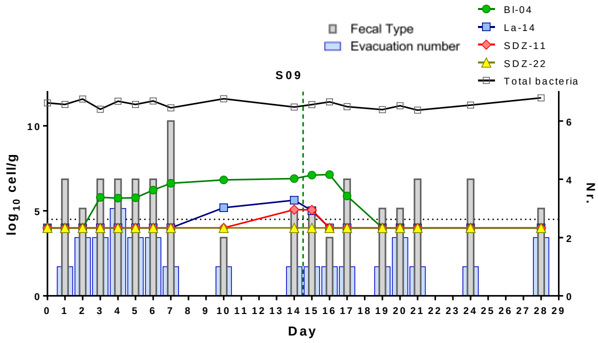
Fecal Type
Evacuation number
Bl-04
La-14
SDZ-11
SDZ-22
Total bacteria
S09
log 10 cell/g
Nr.
Day

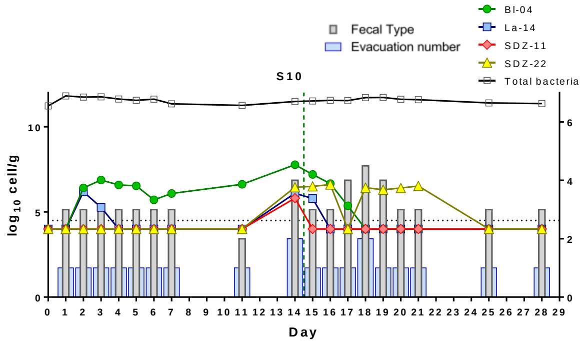
Fecal Type
Evacuation number
Bl-04
La-14
SDZ-11
SDZ-22
Total bacteria
S10
log 10 cell/g
Nr.
Day

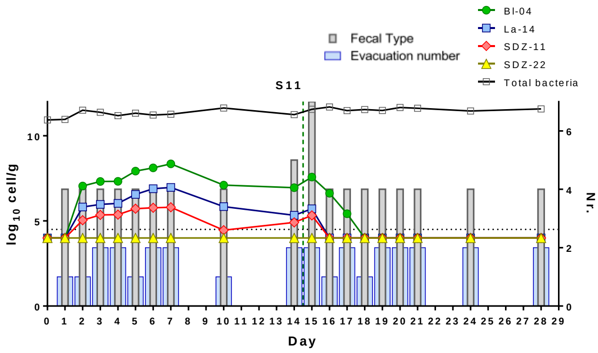
Fecal Type
Evacuation number
Bl-04
La-14
SDZ-11
SDZ-22
Total bacteria
S11
log 10 cell/g
Nr.
Day

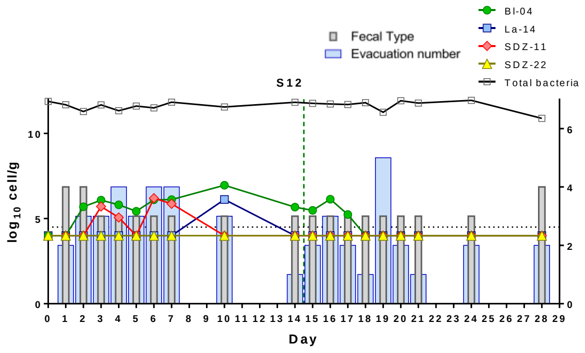
Fecal Type
Evacuation number
Bl-04
La-14
SDZ-11
SDZ-22
Total bacteria
S12
log 10 cell/g
Nr.
Day

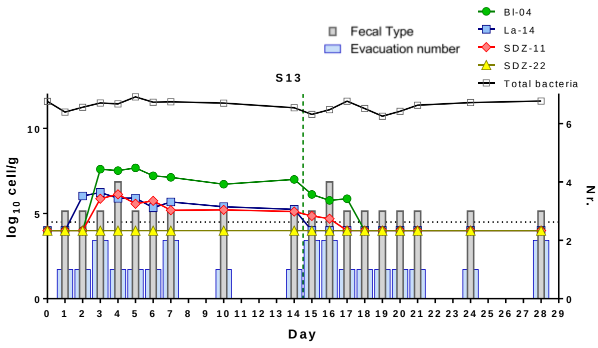
Fecal Type
Evacuation number
Bl-04
La-14
SDZ-11
SDZ-22
Total bacteria
S13
log 10 cell/g
Nr.
Day

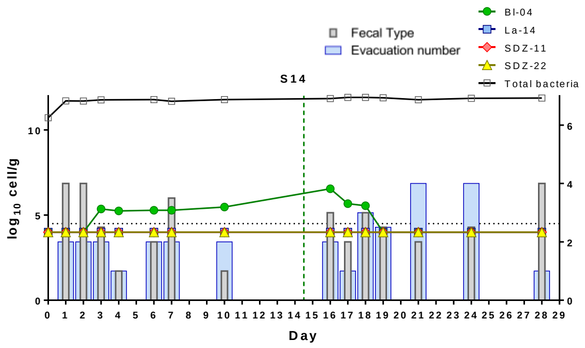
Fecal Type
Evacuation number
Bl-04
La-14
SDZ-11
SDZ-22
Total bacteria
S14
log 10 cell/g
Nr.
Day

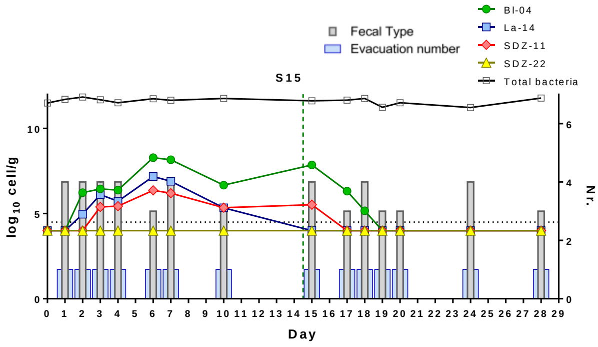
Fecal Type
Evacuation number
Bl-04
La-14
SDZ-11
SDZ-22
Total bacteria
S15
log 10 cell/g
Nr.
Day

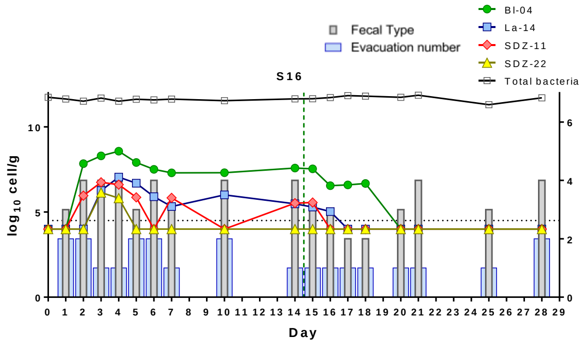
Fecal Type
Evacuation number
Bl-04
La-14
SDZ-11
SDZ-22
Total bacteria
S16
log 10 cell/g
Nr.
Day

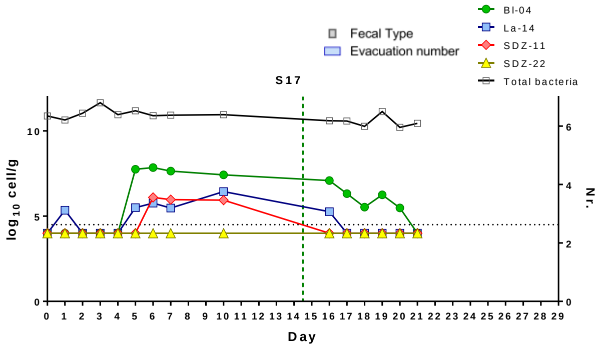
S17
Fecal Type
Evacuation number
Bl-04
La-14
SDZ-11
SDZ-22
Total bacteria
log10 cell/g
Nr.
Day

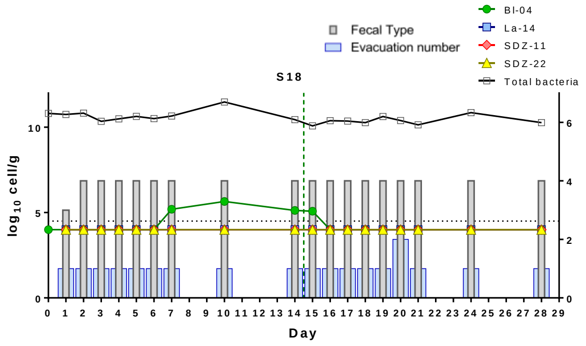
S18
Fecal Type
Evacuation number
Bl-04
La-14
SDZ-11
SDZ-22
Total bacteria
log10 cell/g
Nr.
Day

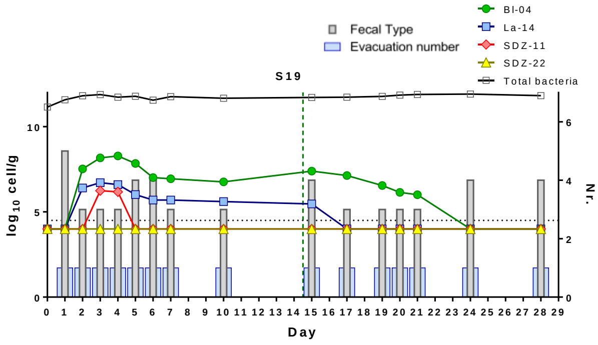
S19
Fecal Type
Evacuation number
Bl-04
La-14
SDZ-11
SDZ-22
Total bacteria
log10 cell/g
Nr.
Day

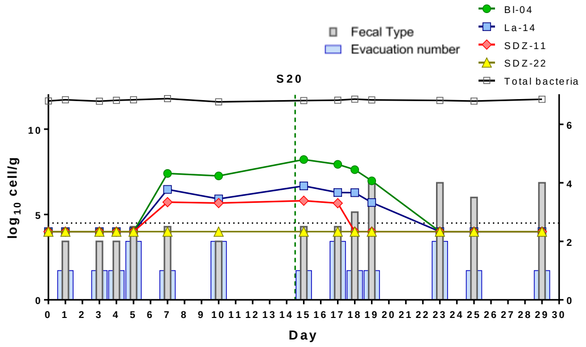
S20
Fecal Type
Evacuation number
Bl-04
La-14
SDZ-11
SDZ-22
Total bacteria
log10 cell/g
Nr.
Day

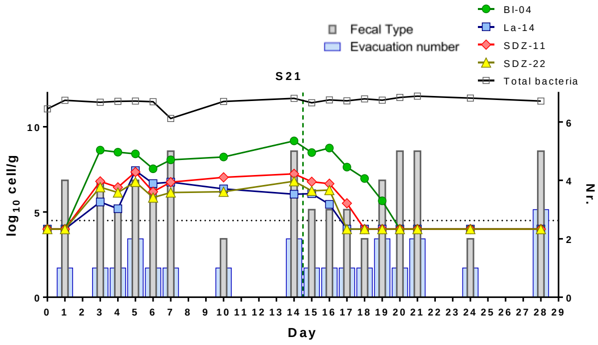
S21
Fecal Type
Evacuation number
Bl-04
La-14
SDZ-11
SDZ-22
Total bacteria
log10 cell/g
Nr.
Day

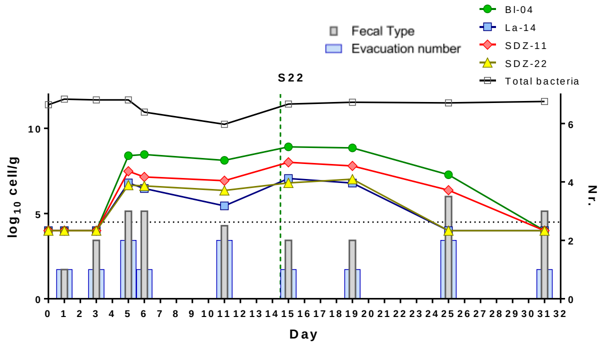
S22
Fecal Type
Evacuation number
Bl-04
La-14
SDZ-11
SDZ-22
Total bacteria
log10 cell/g
Nr.
Day

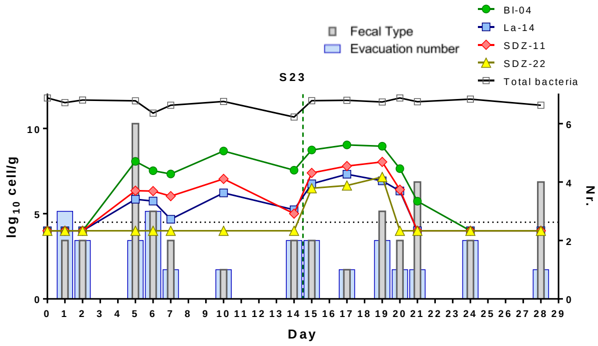
S23
Fecal Type
Evacuation number
Bl-04
La-14
SDZ-11
SDZ-22
Total bacteria
log10 cell/g
Nr.
Day

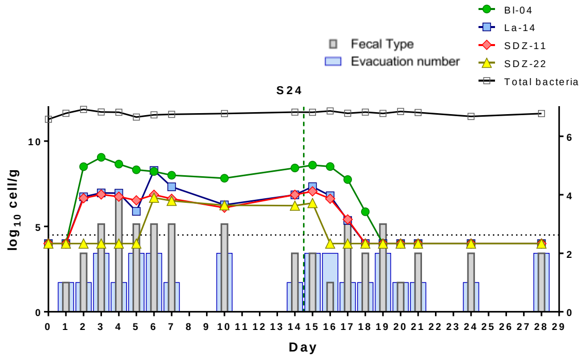
S24
Fecal Type
Evacuation number
Bl-04
La-14
SDZ-11
SDZ-22
Total bacteria
log10 cell/g
Nr.
Day

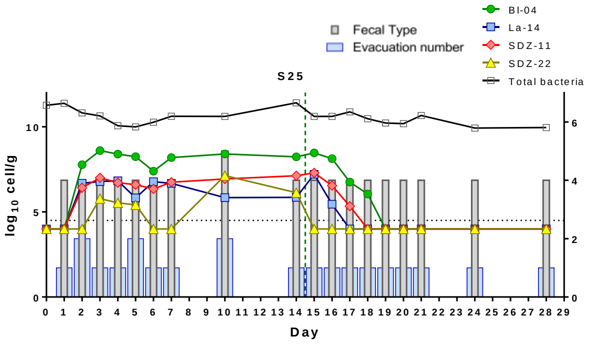
S25
Fecal Type
Evacuation number
Bl-04
La-14
SDZ-11
SDZ-22
Total bacteria
log10 cell/g
Nr.
Day

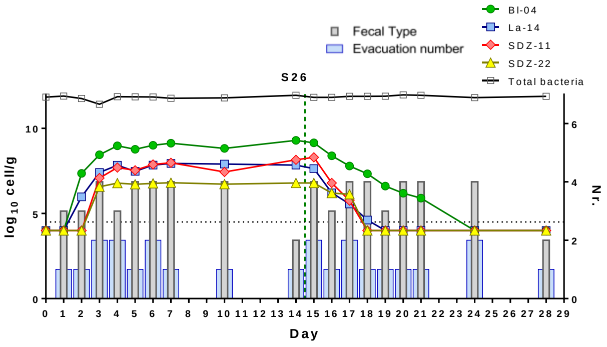
S26
Fecal Type
Evacuation number
Bl-04
La-14
SDZ-11
SDZ-22
Total bacteria
log10 cell/g
Nr.
Day

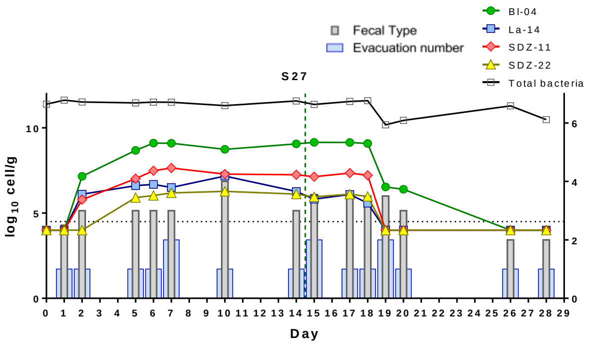
S27
Fecal Type
Evacuation number
Bl-04
La-14
SDZ-11
SDZ-22
Total bacteria
log10 cell/g
Nr.
Day

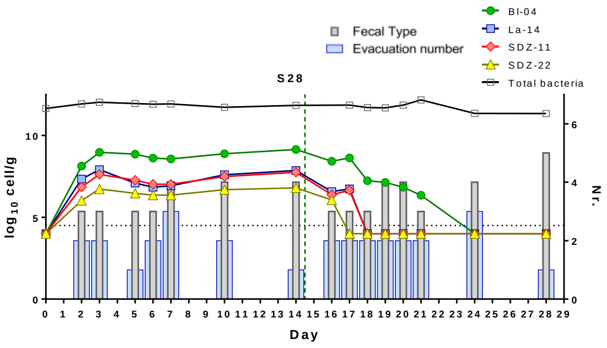
S28
Fecal Type
Evacuation number
Bl-04
La-14
SDZ-11
SDZ-22
Total bacteria
log10 cell/g
Nr.
Day

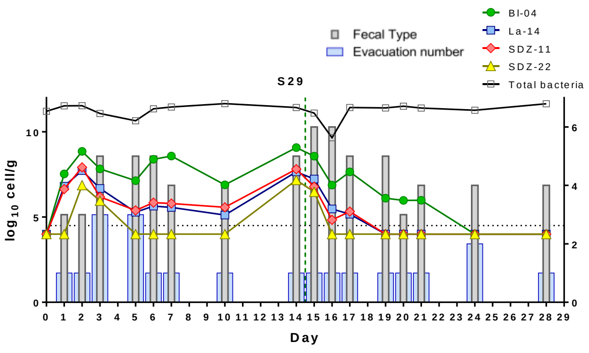
S29
Fecal Type
Evacuation number
Bl-04
La-14
SDZ-11
SDZ-22
Total bacteria
log10 cell/g
Nr.
Day

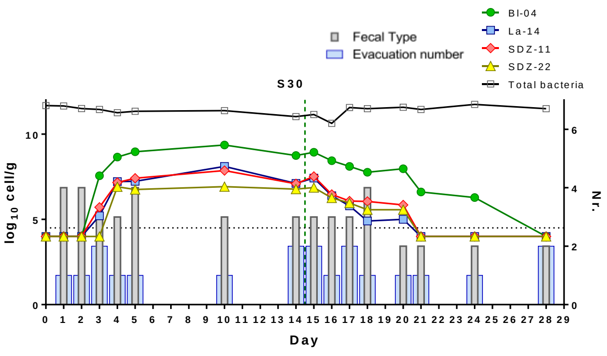
S30
Fecal Type
Evacuation number
Bl-04
La-14
SDZ-11
SDZ-22
Total bacteria
log10 cell/g
Nr.
Day

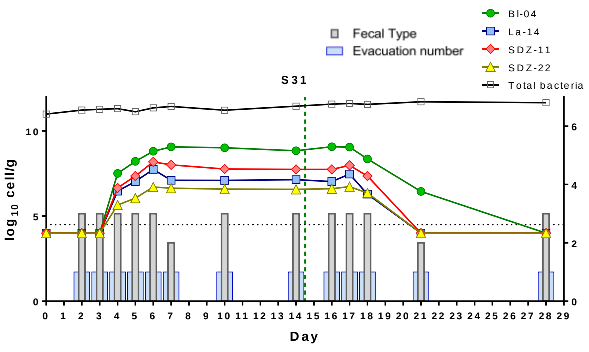
S31
Fecal Type
Evacuation number
Bl-04
La-14
SDZ-11
SDZ-22
Total bacteria
log10 cell/g
Nr.
Day

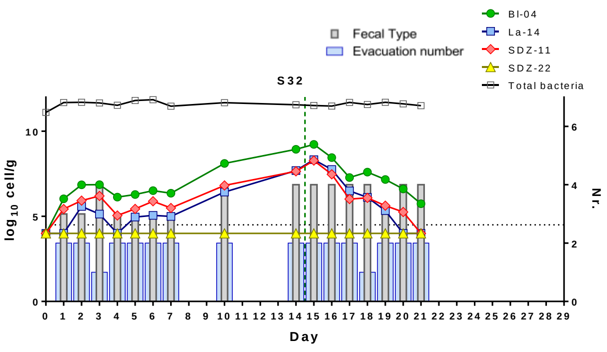
S32
Fecal Type
Evacuation number
Bl-04
La-14
SDZ-11
SDZ-22
Total bacteria
log10 cell/g
Nr.
Day

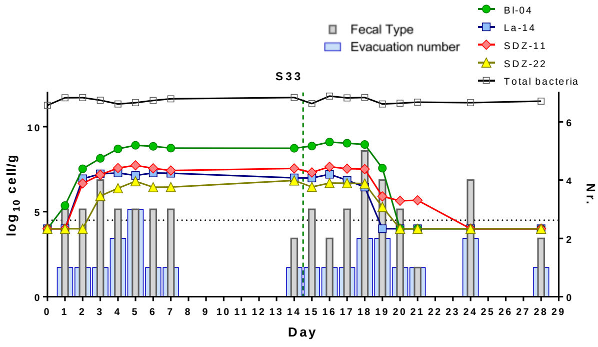
Fecal Type
Evacuation number
Bl-04
La-14
SDZ-11
SDZ-22
Total bacteria
S33
log10 cell/g
Nr.
Day

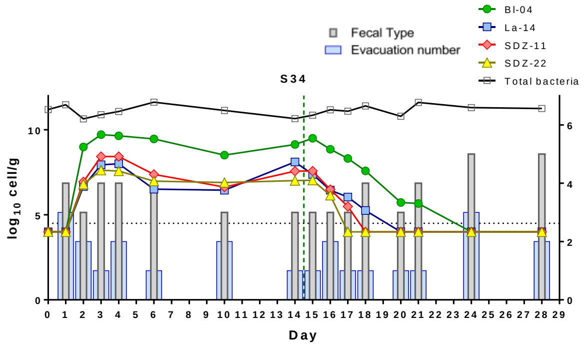
Fecal Type
Evacuation number
Bl-04
La-14
SDZ-11
SDZ-22
Total bacteria
S34
log10 cell/g
Nr.
Day

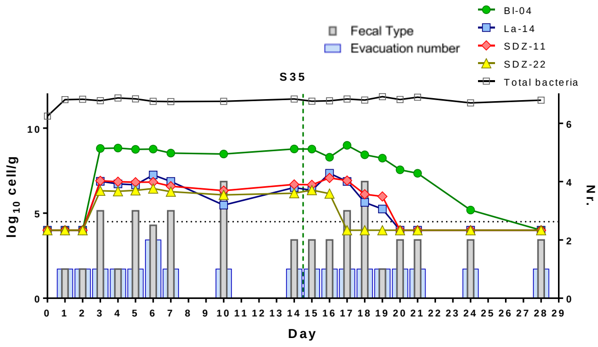
Fecal Type
Evacuation number
Bl-04
La-14
SDZ-11
SDZ-22
Total bacteria
S35
log10 cell/g
Nr.
Day

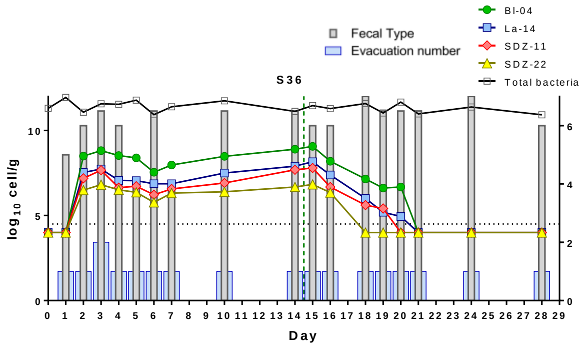
Fecal Type
Evacuation number
Bl-04
La-14
SDZ-11
SDZ-22
Total bacteria
S36
log10 cell/g
Nr.
Day

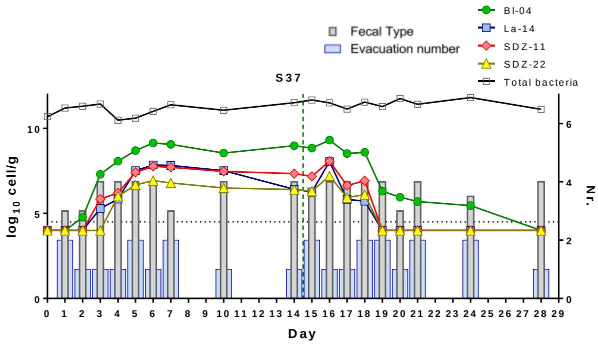
Fecal Type
Evacuation number
Bl-04
La-14
SDZ-11
SDZ-22
Total bacteria
S37
log10 cell/g
Nr.
Day

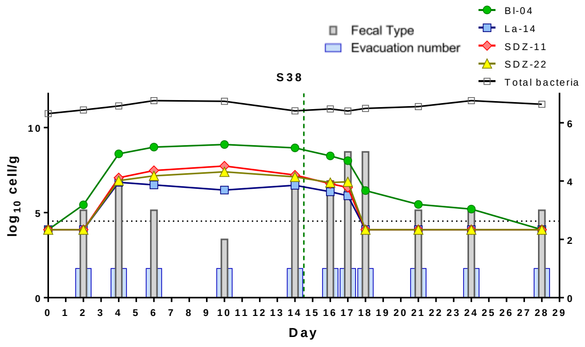
Fecal Type
Evacuation number
Bl-04
La-14
SDZ-11
SDZ-22
Total bacteria
S38
log10 cell/g
Nr.
Day

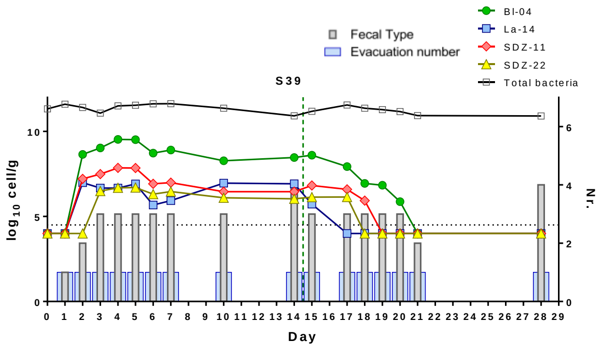
Fecal Type
Evacuation number
Bl-04
La-14
SDZ-11
SDZ-22
Total bacteria
S39
log10 cell/g
Nr.
Day

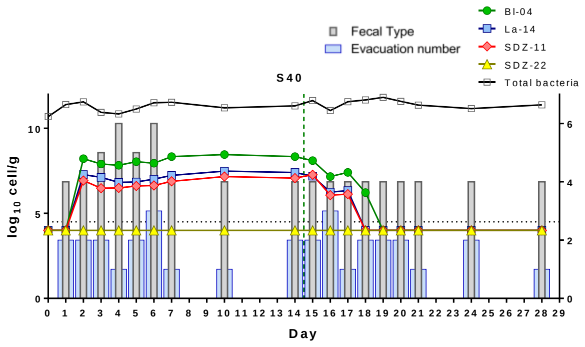
Fecal Type
Evacuation number
Bl-04
La-14
SDZ-11
SDZ-22
Total bacteria
S40
log10 cell/g
Nr.
Day

Figure S3. Isolation of viable cells of the probiotic strains from the feces collected at the end of the intervention period (day 14). Positive recovery is indicated in green. Red boxes indicate that viable cells of a specific probiotic strain have not been isolated from the feces of a specific subject.

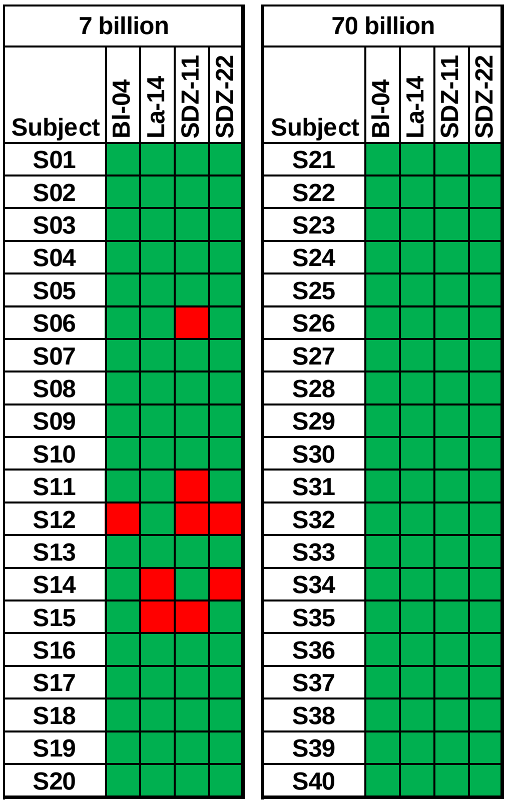

Figure S4. Stability of probiotic cell viability in capsules during the ECoCePPI study as determined through flow cytometer experiments. More details on the flow cytometer protocol adopted are available in the text (material and methods section) and in Figure S1.

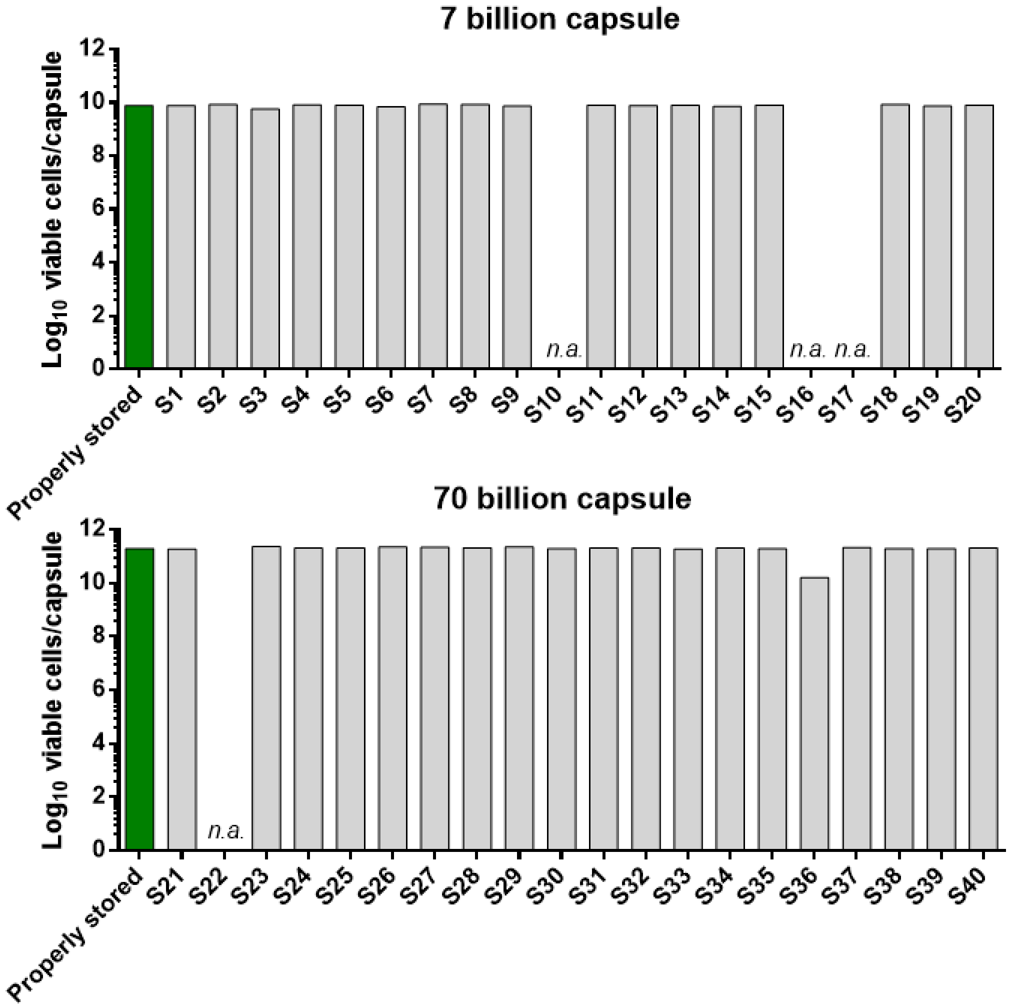

Supplement: Supplementary file 1 [file nutrients-11-00285-s001.pdf]
